# The Knowledge, Concerns, and Beliefs of Mothers Towards Febrile Convulsions and Its Management in Aseer, Saudi Arabia

**DOI:** 10.7759/cureus.71403

**Published:** 2024-10-13

**Authors:** Adel Alawwadh, Danah A Alzahrani, Albaraa M Almallah, Maram S Alshabeeb, Wajan A Alshahrani, Shahad A Alshehri, Lamis S Alshuwayl, Majidah H Halawi, Thekra B Tashari, Lama S Alharbi, Ruya Abdullah, Najlaa Ali, Dania Fatani

**Affiliations:** 1 Pediatrics, Khamis Mushayt Maternity and Children Hospital, Abha, SAU; 2 Medicine and Surgery, King Khalid University, Abha, SAU; 3 College of Medicine, Qassim University, Qassim, SAU; 4 College of Medicine, Jazan University, Jazan, SAU; 5 College of Medicine, Ibn Sina National College, Jeddah, SAU; 6 General Medicine, Arabian Gulf University, Manama, BHR; 7 General Medicine, Jubail General Hospital, Jubail, SAU

**Keywords:** children, febrile convulsions, health education and awareness, mothers' knowledge, saudi arabia

## Abstract

Introduction

Febrile convulsions are common in children aged six months to five years, causing significant parental distress despite being generally harmless. Various beliefs about their causes exist, including high fever and supernatural factors. Previous studies indicate that mothers' education levels and occupations influence their knowledge and attitudes toward febrile convulsions. This study in Aseer, Saudi Arabia, aimed to assess mothers' knowledge, concerns, and beliefs about febrile convulsions to identify educational needs.

Methodology

This cross-sectional study was conducted in Aseer, Saudi Arabia, and performed from October 2023 to July 2024. Data were collected via an online questionnaire. The collected data were cleaned in MS Excel (Redmond, WA: Microsoft Corp.) and analyzed in IBM SPSS version 29 (Armonk, NY: IBM Corp.).

Results

This study surveyed 538 mothers with 25.7% (n=138) aged 36-45 years, and 77.0% (n=414) were married. Regarding the number of children, 25.5% of participants (n=137) had none, 18.6% (n=100) had one child, 14.1% (n=76) had two, and 41.8% (n=225) had three or more children. Education levels showed 56.9% (n=306) had university degrees or higher. Among 401 participants, 30.9% (n=124) reported febrile convulsions in their children. The first convulsion was before one year of age in 14.0% (n=56), and between one and five years in 29.2% (n=117) participants. Misconceptions were common, with 73.3% (n=294) mistakenly believing that febrile convulsions are a form of epilepsy. Higher maternal education was significantly associated with lower knowledge about febrile convulsions (p=0.040), and mothers with children who had experienced febrile convulsions demonstrated significantly lower knowledge (p=0.003).

Conclusion

This study provides valuable insights into the knowledge, concerns, and beliefs of mothers in Aseer, Saudi Arabia, regarding febrile convulsions. While there is a high level of awareness about some aspects, significant misconceptions persist. Higher education and children with febrile convulsions are negative predictors of high awareness and knowledge about febrile convulsions.

## Introduction

Febrile convulsions, also known as febrile seizures, are one of the most common types of seizures observed in children. They are typically associated with a rise in body temperature above 38°C and occur in children between the ages of six months and five years but without evidence of a defined cause [1]. Although febrile convulsions are generally benign and do not cause any long-term harm to children, they can be distressing for parents to witness, it is considered that the parents' distress is comparable to that brought on by a close relative's impending death [2].

Mothers' beliefs about the causes of febrile convulsions varied. Half of the mothers (50%, n=25) attributed febrile convulsions to a high fever (high body temperature), while 30% of parents believed that a supernatural spirit was the cause. Additionally, some parents linked febrile convulsions to witchcraft (24%, n=12), malaria parasites (20%, n=10), stomachache (16%, n=8), and defective brain function (14%, n=7) [3].

In 2014, research was conducted that found a significant association between mothers' level of education and their overall knowledge and attitudes toward febrile convulsions (p=0.00). Sixty-six percent of mothers agreed to administer rectal diazepam themselves if trained. Fifty-six percent of mothers reported gaining their knowledge from friends or family, and 90% expressed a willingness to share their experiences with other mothers [4].

Studies have been published on the knowledge and attitudes regarding febrile seizures among mothers of children under five. Sixty-one percent believed that high temperature in their children could result in seizures, and 63 mothers (65%) thought that this was a life-threatening condition that might lead to brain damage (50%) and disability (50%). Some respondents said they would control seizures by shaking the child (27%), holding the child closely during the seizure (22%), and putting a spoon in the child's mouth (59%). Sixty people (62.5%) prevented febrile seizures by providing them with coffee [5].

In 2021, a study conducted in Al Baha city, showed that 96% of parents agree that fever is harmful to their children and the most common source of information was the internet and pediatricians [6]. A study done in the western region of Saudi Arabia in 2022 showed that working mothers have better knowledge of febrile seizures than housewives. Also, 88.6% of parents answered that “fever can cause convulsion,” while only 4% of parents answered, “convulsion can lead to brain damage” [7].

In Saudi Arabia, specifically in the Aseer region, there is a need to evaluate the knowledge, concerns, and beliefs of mothers regarding febrile convulsions and their management. Understanding the level of awareness in this population is crucial to identifying any knowledge gaps and implementing appropriate educational initiatives.

This cross-sectional study aimed to assess the knowledge, concerns, and beliefs of mothers attending pediatric clinics and primary healthcare facilities in Aseer regarding febrile convulsions and their management. Data will be collected by utilizing surveys/questionnaires to determine the level of knowledge among these mothers and identify areas where education is needed.

## Materials and methods

Study design and sample size

This observational cross-sectional questionnaire survey was conducted in the Aseer region and carried out from October 2023 to July 2024. By using the Raosoft calculator (Seattle, WA: Raosoft, Inc.), the sample size was estimated, with a confidence level of 95% and a marginal error of 5%, the minimal sample size was 384. The formula for calculating sample size is as follows: 

$n=\frac{n&times;Z^{2}&times;p&times;(1-p)}{E^{2}&times;(n-1)+Z^{2}&times;p&times;(1-p)}$

Here, n is the population size, Z is the Z-value (for 95% confidence, Z≈1.96 ), p is the estimated proportion of the population (response distribution), and E is the margin of error (as a decimal).

Given that n = 2,024,285, E = 0.05 (5% margin of error), p = 0.5 (50% response distribution), Z = 1.96 (for 95% confidence level). The required sample size is 384 to achieve a 5% margin of error at a 95% confidence level with a population of 2,024,285 and a response distribution of 50%. This was calculated as follows:

$n = \frac{2024285 &times; (1.96^{2}) &times; 0.5 &times; (1-0.5)}{(0.05^{2}) &times; (2024285-1) + (1.96^{2}) &times; 0.5 &times; (1-0.5)}$

$n = \frac{2024285 &times; 3.8416 &times; 0.25}{0.0025 &times; 2024284 + 3.8416 &times; 0.25}$

$n \approx \frac{1940806.72}{5061.67} \approx 383.36$

Thus, n≈384

Inclusion and exclusion criteria

The inclusion criteria for the study were Saudi mothers aged 18 years and above who reside in the Aseer region of Saudi Arabia, are able to give informed consent, are fluent in Arabic (as the survey and study materials are provided in Arabic), and have at least one child (biological or adopted). The exclusion criteria included non-Saudi mothers or those residing outside the Aseer region, mothers below the age of 18 years, those unable to give informed consent, non-Arabic speakers, and mothers without children.

Method for data collection

The study utilized a self-administered, structured questionnaire adapted from a previously published article, with consent obtained from the primary author [7]. The questionnaire was hosted on Google Forms (Mountain View, CA: Google LLC), which was distributed via social media channels to reach the target population. The questionnaire was organized into three main sections as follows: questionnaire 1 assessed sociodemographic parameters with seven closed-ended multiple-choice questions focusing on age, marital status, number of children, and parent's education and occupation (appendix 1); questionnaire 2 included nine closed-ended questions related to the features and prevalence of febrile convulsions, encompassing the child’s experience with seizures, the number of infections per year, and family history (appendix 2); questionnaire 3 evaluated awareness and management of febrile convulsions with 16 multiple-choice questions addressing knowledge, beliefs, and misconceptions. In total, the questionnaire comprised 32 questions (appendix 3). To ensure cultural relevance and clarity, the questionnaire was reviewed by pediatricians and public health experts in the Aseer region, with a focus on linguistic adaptation and appropriateness for the local population. Additionally pilot study involving 50 participants was conducted to assess the clarity and functionality of the survey, leading to minor revisions for improved readability and comprehension.

Statistical analysis

Data were entered into MS Excel (2016) for Windows. Following data entry, the dataset was transferred to the SPSS version 29.0.0 (Armonk, NY: IBM Corp.) for statistical analysis. A comprehensive statistical analysis was conducted on the dataset, utilizing both descriptive and inferential methodologies. Initially, descriptive statistics were computed to summarize the demographic characteristics of the participants, including age, gender, and other relevant features, providing an overview of the study population. To assess the association between knowledge levels and various sociodemographic factors, chi-square and Fisher's exact tests were utilized. Subsequently, binary logistic regression analysis was employed to determine the predictors of high knowledge levels regarding febrile convulsions. In this study, a high knowledge level was defined as a score above the 50th percentile on the knowledge assessment section of the questionnaire. Statistical significance was established at a p-value of 0.05 or lower, and a 95% confidence interval was used to assess the reliability of the estimates. All statistical analyses were conducted using IBM SPSS software, version 29.0.0.

## Results

This study included 538 mothers assessed for febrile convulsions. Notably, the age distribution shows that 13.9% (n=75) were aged 18-25 years, 18.8% (n=101) were aged 26-35 years, 25.7% (n=138) were aged 36-45 years, and 16.2% (n=87) were over 45 years. Most participants were married (77.0%, n=414), with a smaller portion being single (20.1%, n=108) or widowed/divorced (3.0%, n=16). Regarding the number of children, 25.5% (n=137) had no children, 18.6% (n=100) had one child, 14.1% (n=76) had two children, and 41.8% (n=225) had three or more children. Mothers’ education levels were predominantly at the university level or higher (56.9%, n=306), while 45.7% (n=246) were employed. Fathers' education also leaned toward university level or higher (54.6%, n=294), with 59.1% (n=318) employed (Table 1).

**Table 1:** Sociodemographic parameters of participants.

Variables		Frequency (n=538)	Percent
Age	18-25 years	75	13.9%
	26-35 years	101	18.8%
	36-45 years	138	25.7%
	>45 years	87	16.2%
Marital status	Widow/divorced	16	3.0%
	Single	108	20.1%
	Married	414	77.0%
No. of children	0	137	25.5%
	1	100	18.6%
	2	76	14.1%
	≥3	225	41.8%
Education of mother	Below secondary school	21	3.9%
	Secondary school	74	13.8%
	University/higher	306	56.9%
Occupation of mother	Housewife	155	28.8%
	Working	246	45.7%
Education of father	Below secondary school	26	4.8%
	Secondary school	81	15.1%
	University/higher	294	54.6%
Occupation of father	Unemployed/retired	83	15.4%
	Employee/work	318	59.1%

Table 2 shows the different febrile convulsions and their prevalence among children. Out of 401 participants, 30.9% (n=124) reported that their children had experienced febrile convulsions, while 69.1% (n=277) had not. The first febrile seizure occurred in 14.0% (n=56) of children before one year of age, in 29.2% (n=117) between one and five years, and in 4.7% of children (n=19) after five years. Among the children, 28.2% (n=113) were male, and 23.7% (n=95) were female. Regarding infections per year, 29.9% (n=120) had one infection, while only a small percentage had more frequent infections. Most siblings (88.0%, n=353) and family members (85.8%, n=344) did not experience febrile convulsions. Concerning information sufficiency, 45.6% (n=183) of mothers felt they lacked sufficient information, 45.6% (n=183) felt they had some but not enough information, and only 8.7% (n=35) felt adequately informed. When their child had a fever, 94.8% (n=380) administered antipyretic medicine and 92.5% (n=371) used a medical thermometer to measure the child’s temperature.

**Table 2:** Different features related to febrile convulsions of child and its prevalence. *Mothers who reported having one or more children.

Variables		Frequency (n=401)*	Percent
Have any of your children ever had febrile convulsions?	No	277	69.1%
	Yes	124	30.9%
The child’s age when the first febrile seizure occurred	<1 years	56	14.0%
	1-5 years	117	29.2%
	>5 years	19	4.7%
Sex of child	Female	95	23.7%
	Male	113	28.2%
Number of infections per year	1 time	120	29.9%
	2 times	46	11.5%
	3 times	12	3.0%
	>3 times	13	3.2%
Number of febrile convulsions in siblings?	0	353	88.0%
	1	31	7.7%
	2	16	4.0%
	≥3	1	0.2%
Number of febrile convulsions in family members?	0	344	85.8%
	1	40	10.0%
	2	9	2.2%
	≥3	8	2.0%
Do you have sufficient information about febrile convulsions?	No	183	45.6%
	Yes (but not enough)	183	45.6%
	Yes (enough)	35	8.7%
Do you give antipyretic medicine at home when your child has a fever?	No	21	5.2%
	Yes	380	94.8%
Do you use a medical thermometer at home to measure your child’s temperature if he has a fever?	No	30	7.5%
	Yes	371	92.5%

Table 3 shows the awareness of mother about febrile convulsions and their management. Notably, 95.5% (n=383) correctly identified that fever can cause convulsions. However, 73.3% (n=294) incorrectly believed febrile convulsions are epilepsy. A significant percentage, 89.5% (n=359), correctly stated that febrile convulsions between three months and five years are risky, and 64.8% (n=260) correctly noted that it is common at this age. On the other hand, 69.3% (n=278) incorrectly believed recurrent fever does not increase the risk. Most (92.3%, n=370) participants understood that higher fever increases the risk. A total of 70.8% (n=284) correctly stated febrile convulsions can progress to epilepsy, while 54.9% (n=220) identified developmental delay as a risk factor. Moreover, 55.4% (n=222) incorrectly believed that family history has no effect on risk, and 89.5% (n=359) knew febrile convulsions were not fatal. Additionally, 67.8% (n=272) correctly stated children can be vaccinated on schedule, and 85.8% (n=344) agreed that EEG or CT scans are necessary. Misconceptions included the need for a protective device during convulsions (63.1%, n=253) and mouth-to-mouth resuscitation (61.1%, n=245). Lastly, 84.3% (n=338) correctly noted that not all children need medication, and 55.9% (n=224) recognized that traditional medication is unnecessary.

**Table 3:** Awareness of participants about febrile convulsions and its management.

Variables		Frequency (n=401)	Percent
Fever can cause convulsions	Incorrect	18	4.5%
	Correct	383	95.5%
Febrile convulsion is epilepsy	Incorrect	294	73.3%
	Correct	107	26.7%
Febrile convulsion at the age of 3 months to 5 years is risky	Incorrect	42	10.5%
	Correct	359	89.5%
Febrile convulsion is common at the age of 5 years	Incorrect	141	35.2%
	Correct	260	64.8%
Recurrent fever dose not increase the risk of febrile convulsion	Incorrect	278	69.3%
	Correct	123	30.7%
Higher fever increases the risk of febrile convulsion	Incorrect	31	7.7%
	Correct	370	92.3%
Febrile convulsion can progress to epilepsy	Incorrect	117	29.2%
	Correct	284	70.8%
Developmental delay increases the risk of febrile convulsion	Incorrect	181	45.1%
	Correct	220	54.9%
Family history of convulsive disorder has no effect on the risk of febrile convulsion	Incorrect	222	55.4%
	Correct	179	44.6%
Febrile convulsion is fatal	Incorrect	42	10.5%
	Correct	359	89.5%
Children with febrile convulsion can receive vaccination on schedule	Incorrect	129	32.2%
	Correct	272	67.8%
Electroencephalogram or computer tomography is necessary in children with febrile convulsion	Incorrect	57	14.2%
	Correct	344	85.8%
It is not necessary to put a protective device into the mouth to prevent tongue injury during convulsion	Incorrect	253	63.1%
	Correct	148	36.9%
It is necessary to do mouth-to-mouth resuscitation during convulsion	Incorrect	245	61.1%
	Correct	156	38.9%
Medications are needed for every child with febrile convulsion	Incorrect	63	15.7%
	Correct	338	84.3%
Traditional medication is necessary as therapy	Incorrect	224	55.9%
	Correct	177	44.1%

**Figure 1:**
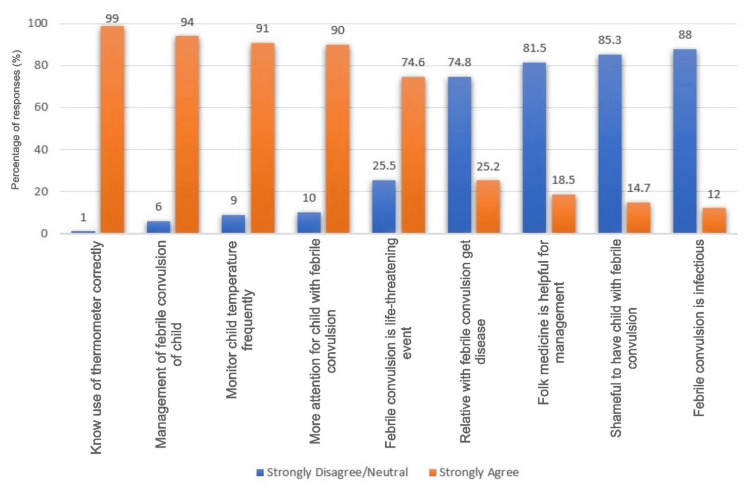
Concerns and believes of mother about febrile convulsion.

Figure 1 shows the mothers' concerns and beliefs about febrile convulsions. Most mothers (99%) strongly agreed that they know how to use a thermometer correctly, and 94% felt confident in managing febrile convulsions. Additionally, 91% frequently monitored their child's temperature, and 90% believed that more attention was needed for children with febrile convulsions. However, 74.6% viewed febrile convulsions as life-threatening, while 25.2% thought that relatives with febrile convulsions were more likely to get the disease. A smaller proportion of mothers had less scientifically supported beliefs. Specifically, 18.5% believed in the efficacy of folk medicine for managing febrile convulsions, 14.7% felt that it was shameful to have a child with febrile convulsions, and 12% considered febrile convulsions to be infectious.

Table 4 shows the association between the knowledge level of mothers with different features. Notably, age did not show a significant association with knowledge level (p=0.252), with knowledge being relatively consistent across age groups. Marital status also did not significantly affect knowledge, as 26.5% of married mothers and 13.3% of single, widowed, or divorced mothers had high knowledge (p=0.372). The number of children and mothers' education level showed no significant association, with high knowledge percentages of 31.0%, 25.0%, and 24.4% for mothers with one, two, and three or more children, respectively (p=0.448), and 24.8% for those with university education or higher (p=0.365). Mothers' occupation did not influence knowledge level (p=0.742), with 27.1% of housewives and 25.6% of working mothers having high knowledge. Fathers' education and occupation also showed no significant association (p=0.652 and p=0.837, respectively). The presence of a child with febrile convulsions did not significantly correlate with knowledge level (p=0.112), nor did the presence of siblings or family members with febrile convulsions (p=0.616 and p=0.981, respectively).

**Table 4:** Association between knowledge level of mothers and different features. *Chi-square test. **Fisher’s exact test.

Variables			Knowledge about febrile convulsions		Significant value
			Low level (<50th percentile)	High level (>50th percentile)	
Age	18-25 years	N	51	24	0.252^*^
		%	68.0%	32.0%	
	26-35 years	N	77	24	
		%	76.2%	23.8%	
	36-45 years	N	108	30	
		%	78.3%	21.7%	
	>45 years	N	60	27	
		%	69.0%	31.0%	
Marital status	Single/widow/divorced	N	13	2	0.372^**^
		%	86.7%	13.3%	
	Married	N	283	102	
		%	73.5%	26.5%	
No. of child	1 Child	N	69	31	0.448^*^
		%	69.0%	31.0%	
	2 Children	N	57	19	
		%	75.0%	25.0%	
	≥3 Children	N	170	55	
		%	75.6%	24.4%	
Education of mother	Less than secondary school	N	13	8	0.365^*^
		%	61.9%	38.1%	
	Secondary school	N	53	21	
		%	71.6%	28.4%	
	University/higher	N	230	76	
		%	75.2%	24.8%	
Occupation of mother	Housewife	N	113	42	0.742^*^
		%	72.9%	27.1%	
	Working	N	183	63	
		%	74.4%	25.6%	
Education of father	Less than secondary school	N	21	5	0.652^*^
		%	80.8%	19.2%	
	Secondary school	N	58	23	
		%	71.6%	28.4%	
	University/higher	N	217	77	
		%	73.8%	26.2%	
Occupation of father	Unemployed/retired	N	62	21	0.837^*^
		%	74.7%	25.3%	
	Employee/work	N	234	84	
		%	73.6%	26.4%	
Any child with febrile convulsion	No	N	198	79	0.112^*^
		%	71.5%	28.5%	
	Yes	N	98	26	
		%	79.0%	21.0%	
Any sibling with febrile convulsion	No	N	262	91	0.616^*^
		%	74.2%	25.8%	
	Yes	N	34	14	
		%	70.8%	29.2%	
Any family member with febrile convulsion	No	N	254	90	0.981^*^
		%	73.8%	26.2%	
	Yes	N	42	15	
		%	73.7%	26.3%	

Table 5 shows that the binary logistic regression model identified several factors influencing the high knowledge level of mothers regarding febrile convulsions. Age and marital status were not significant predictors, as were the number of children and the father's education and occupation. Interestingly, higher education among mothers was significantly associated with lower knowledge levels (regression coefficient B=-0.823, p=0.040; exponentiation of the B coefficient, Exp(B)=0.439). Conversely, working mothers tended to have higher knowledge levels, though this was marginally significant (B=0.758, p=0.067, Exp(B)=2.134). Another key finding was that mothers with children who had febrile convulsions were less likely to have high knowledge levels (B=-1.078, p=0.003, Exp(B)=0.340). The number of infections per year, febrile convulsions in siblings, and family members were not significant predictors. Additionally, having sufficient information about febrile convulsions did not significantly influence the knowledge level.

**Table 5:** Adjusted predictors of high knowledge level among mothers (binary logistic regression model).

Variables	B	Significance	Exp(B)	95% CI	
				Lower	Upper
Age	-0.045	0.859	0.956	0.581	1.574
Marital status (married)	0.235	0.590	1.265	0.539	2.968
Number of children	-0.403	0.206	0.669	0.358	1.248
Higher education of mother	-0.823	0.040	0.439	0.200	0.964
Mother’s occupation (working women)	0.758	0.067	2.134	0.947	4.806
Higher education of father	0.376	0.313	1.457	0.701	3.026
Father’s occupation (employed)	0.244	0.607	1.276	0.504	3.233
Children with febrile convulsion	-1.078	0.003	0.340	0.166	0.699
Number of infections/year	-0.191	0.399	0.826	0.529	1.289
Febrile convulsions in siblings	-0.097	0.799	0.908	0.430	1.914
Febrile convulsions in family members	0.075	0.793	1.078	0.616	1.885
Sufficient information about febrile convulsions?	-0.042	0.828	0.959	0.660	1.395
Constant	0.192	0.916	1.211	-	-

## Discussion

Febrile convulsions, common in children aged six months to five years, are associated with high body temperature and can cause significant parental distress despite being generally benign [8]. A study by Tiwari et al. shows that according to the NIH consensus conference, febrile seizure is a condition that typically affects children aged three months to five years [9]. Mothers hold varied beliefs about their causes, including high fever, supernatural spirits, witchcraft, and more. Similarly, a study by Owusu shows that participants attributed three major causes of febrile seizures as follows: natural/biological, social, and spiritual [10]. Previous studies show that mothers' education and occupation influence their knowledge and attitudes toward febrile convulsions [11]. Our cross-sectional study in Aseer, Saudi Arabia, aimed to assess mothers' knowledge, concerns, and beliefs about febrile convulsions and their management by surveying those attending pediatric clinics and primary healthcare facilities, identifying knowledge gaps, and guiding educational initiatives.

Notably, our study shows that a significant portion of the participants were aged 36-45 years (25.7%). The majority were married (77.0%), and a substantial number had three or more children (41.8%). Most mothers had a university or higher education (56.9%), and a significant proportion were working (45.7%). These demographic factors are essential as they provide context for understanding the knowledge and beliefs about febrile convulsions.

Notably, febrile convulsions were reported in 30.9% of the children. This prevalence is consistent with findings from other regions where febrile seizures are common in pediatric populations. However, a study by Alhumaidy et al. shows that the prevalence of febrile convulsions among children in Saudi Arabia was 6.8% [12]. Most first febrile seizures occurred between one and five years of age, aligning with the known peak age for febrile convulsions. However, a study by Xixis et al. shows that febrile seizures have an incidence of 2-5% among children in the United States and Europe, peaking between 12 months (one year) and 18 months (1.5 years) of age [13]. Moreover, our study showed that a higher percentage of male children (28.2%) experienced febrile convulsions compared to female children (23.7%), which is consistent with existing literature suggesting a slight male predominance in febrile convulsions. A study by Gourabi et al. shows that more cases of febrile seizures were in boys (57.9%) as compared to girls (42.1%) [14].

Notably, a high level of awareness was observed among mothers regarding certain aspects of febrile convulsions. For instance, 95.5% correctly identified that fever could cause convulsions, and 89.5% understood that febrile convulsions between three months and five years are risky [15]. However, significant misconceptions persisted. Notably, 73.3% mistakenly believed that febrile convulsions are a form of epilepsy, and 69.3% incorrectly thought that recurrent fever does not increase the risk of febrile convulsions. These misconceptions highlight the need for better educational interventions. Comparing our findings with previous studies, a study by Schmitt in Egypt found similar misconceptions, with many parents believing febrile seizures were a form of epilepsy and underestimating the risks associated with recurrent fevers [16]. This suggests a broader issue of misinformation that extends beyond our study population. Similarly, various misconceptions about febrile convulsions are highlighted by Huang et al. about FC, including folk medicines, brain damage, and subsequent epilepsy, and mitigating the parents' anxieties [17].

Moreover, our study shows that the concerns and beliefs of mothers about febrile convulsions reflect both accurate knowledge and significant misconceptions. A majority of mothers felt confident in managing febrile convulsions and frequently monitored their child's temperature. However, a study by Sajadi et al. shows that FC was particularly worrisome for parents and they often required information (due to less information about the management of FC) that could enable them to manage their child’s illness [18]. Notably, 74.6% viewed febrile convulsions as life-threatening, which might lead to unnecessary anxiety and overreaction. This is contrasted by a study in Germany by Rice et al., which found that while parents were concerned about febrile seizures, only 20% considered them life-threatening [19]. This disparity could be due to cultural differences in health perceptions or differences in the healthcare information provided to parents. Interestingly, a small proportion of mothers believed in the efficacy of folk medicine (18.5%) and considered febrile convulsions to be infectious (12%). These beliefs were less common in studies conducted in more urbanized or developed regions, suggesting that educational level and access to healthcare information might influence these misconceptions [20].

Notably, the regression analysis conducted in our study identified higher education among mothers as significantly associated with lower knowledge levels about febrile convulsions. This counterintuitive finding might be explained by the possibility that more educated mothers rely on less credible sources of information, such as the Internet, rather than professional medical advice. This contrasts with findings from a study by Alotaibi et al. in the Kingdom of Saudi Arabia, which showed that higher education levels were associated with better understanding and management of febrile convulsions. This study also identifies age, marital status, gender, residence, occupation, and number of children as significant predictors of knowledge about febrile convulsions [21]. This discrepancy emphasizes the importance of tailored health education programs that address the specific information needs and sources used by different educational groups. Moreover, working mothers tended to have higher knowledge levels, though this was marginally significant. This finding aligns with the hypothesis that working mothers might have better access to healthcare information through workplace health programs or better socio-economic conditions allowing access to private healthcare. Interestingly, mothers with children who had experienced febrile convulsions were less likely to have high knowledge levels. This finding may indicate that personal experience alone is insufficient for comprehensive understanding and that these mothers may benefit from targeted education and support.

Limitations

There are several limitations of this study including a potentially non-representative sample due to regional focus, reliance on self-reported data which may introduce bias, and limited generalizability of findings beyond the Aseer region. Additionally, the cross-sectional design limits causal inferences, and some demographic subgroups were underrepresented, affecting the robustness of conclusions.

Implications and future direction

Our study highlights the need for comprehensive health education programs tailored to the specific needs of mothers in the Aseer region. Such programs should address common misconceptions, provide clear information about the risks and management of febrile convulsions, and be accessible to all socio-demographic groups. Healthcare providers should be trained to deliver consistent and accurate information to parents, potentially through routine pediatric visits or community health workshops.

## Conclusions

This study highlights that while mothers in the Aseer region possess a general awareness of febrile convulsions, significant misconceptions and knowledge gaps persist, particularly in differentiating febrile convulsions from epilepsy and understanding proper management techniques. The finding that higher education levels correlate with increased misconceptions suggests that even well-educated mothers may lack accurate information. These gaps can lead to unnecessary anxiety and improper responses during convulsive episodes. Therefore, there is a critical need for healthcare providers to offer targeted educational interventions that address these specific misconceptions, ensuring that parents are better equipped to manage febrile convulsions, ultimately improving child health outcomes.

## Appendices

Appendix 1

**Table 6:** Sociodemographic parameters of participants.

Variables	Values
Age	18-25 years
	26-35 years
	36-45 years
	More than 45 years
Marital status	Widow/divorced/separated
	Married
No. of children	0
	1
	2
	≥3
Education of mother	Below secondary school
	Secondary school
	University/higher
Occupation of mother	Housewife
	Working
Education of father	Below secondary school
	Secondary school
	University/higher
Occupation of father	Unemployed/retired
	Employee/work

Appendix 2

**Table 7:** Different features related to febrile convulsions of child and its prevalence.

Variables	Values
Have any of your children ever had febrile convulsions?	No
	Yes
The child’s age when the first febrile seizure occurred	<1 years
	1-5 years
	>5 years
Sex of child	Female
	Male
Number of infections per year	1 time
	2 times
	3 times
	>3 times
Number of febrile convulsions in siblings?	0
	1
	2
	≥3
Number of febrile convulsions in family members?	0
	1
	2
	≥3
Do you have sufficient information about febrile convulsions?	No
	Yes (but not enough)
	Yes (enough)
Do you give antipyretic medicine at home when your child has a fever?	No
	Yes
Do you use a medical thermometer at home to measure your child’s temperature if he has a fever?	No
	Yes

Appendix 3

**Table 8:** Awareness of participants about febrile convulsions and their management.

Variables	Values
Fever can cause convulsions	Incorrect
	Correct
Febrile convulsion is epilepsy	Incorrect
	Correct
Febrile convulsion at the age of 3 months to 5 years is risky	Incorrect
	Correct
Febrile convulsion is common at the age of 5 years	Incorrect
	Correct
Recurrent fever dose not increase the risk of febrile convulsion	Incorrect
	Correct
Higher fever increases the risk of febrile convulsion	Incorrect
	Correct
Febrile convulsion can progress to epilepsy	Incorrect
	Correct
Developmental delay increases risk of febrile convulsion	Incorrect
	Correct
Family history of convulsive disorder has no effect on the risk of febrile convulsion	Incorrect
	Correct
Febrile convulsion is fatal	Incorrect
	Correct
Children with febrile convulsion can receive vaccination on schedule	Incorrect
	Correct
Electroencephalogram or computer tomography is necessary in child with febrile convulsion	Incorrect
	Correct
It is not necessary to put a protective device into the mouth to prevent tongue injury during convulsion	Incorrect
	Correct
It is necessary to do mouth to mouth resuscitation during convulsion	Incorrect
	Correct
Medications are needed for every child with febrile convulsion	Incorrect
	Correct
Traditional medication is necessary as therapy	Incorrect
	Correct
